# Application of Machine Learning Algorithms in Estimating Live Weight of Yucatecan Criollo Pigs Through Biometric Measurements

**DOI:** 10.3390/ani16081134

**Published:** 2026-04-08

**Authors:** Angel C. Sierra-Vasquez, Cem Tırınk, Jesus A. Mezo-Solis, Hasan Önder, Naomi Cih-Angulo, Uğur Şen, Julio C. Rodriguez-Perez, Jorge C. Bojorquez-Cat, Kadyrbai Chekirov, İsa Coşkun, Alfonso Juventino Chay-Canul

**Affiliations:** 1 National Technological Institute of Mexico Campus Conkal, Conkal 97345, Yucatán, Mexico; angel.sierra@itconkal.edu.mx (A.C.S.-V.); julio.perez@itconkal.edu.mx (J.C.R.-P.);; 2 Department of Animal Science, Faculty of Agriculture, Iğdır University, Igdır TR76000, Türkiye; cem.tirink@igdir.edu.tr; 3 Department of Animal Science, Faculty of Agriculture, Ondokuz Mayıs University, Samsun TR55139, Türkiye; 4 Department of Agricultural Biotechnology, Faculty of Agriculture, Ondokuz Mayıs University, Samsun TR55139, Türkiye; ugur.sen@omu.edu.tr; 5 Department of Biology, Faculty of Science, Kyrgyz-Turkish Manas University, Bishkek 720038, Kyrgyzstan; kadyr.chekirov@manas.edu.kg; 6 Department of Animal Science, Faculty of Agriculture, Kırşehir Ahi Evran University, Kırşehir TR40100, Türkiye; isa.coskun@ahievran.edu.tr; 7 División Académica de Ciencias Agropecuarias, Universidad Juárez Autónoma de Tabasco, Villahermosa 86040, Tabasco, Mexico; alfonso.chay@ujat.mx

**Keywords:** XGBoost, LightGBM, live weight, prediction, machine learning

## Abstract

This study compares the performance of XGBoost and LightGBM models for predicting live weights of Yucatecan Criollo pigs from biometric measurements and examines the structural and algorithmic differences that affect model fit. Detailed analysis of the models’ hyperparameter optimization and variable importance revealed how each model approaches the data and prioritizes features. This study was conducted on 182 Yucatecan Criollo pigs (134 females and 48 males). When model performances were evaluated, the XGBoost model showed superior prediction performance with acceptable accuracy and lower error rates in the test dataset, with R^2^ = 0.905, RMSE = 5.704, and MAE = 3.636. In contrast, the LightGBM model produced acceptable results under certain hyperparameter combinations with R^2^ = 0.824, RMSE = 7.772, and MAE = 5.505. While the robust performance of both models requires strategic decisions in model selection and optimization, it is recommended to consider the dataset’s nature in feature selection and hyperparameter settings. This study provides important insights for simplifying the model and improving its efficiency in machine learning applications, and serves as a reference for more effective model use.

## Simple Summary

This research leverages artificial intelligence to estimate the live weight of Yucatecan Criollo pigs using only body measurements (height, width, etc.). This study compares the performance of XGBoost and LightGBM, two gradient-boosting machine learning algorithms widely used in the literature. Researchers examined how accurately these “digital brains” predicted weight and which measurements they prioritized when making decisions. The results show that the XGBoost model stood out in weight estimation, particularly with its high accuracy rates, while LightGBM provided quite fast and effective results with specific adjustments. The most important finding of the study is that there is no single “best” model; the most accurate results require selecting settings appropriate to the dataset’s structure. This work suggests that artificial intelligence in livestock farming can be simplified and made more efficient, transforming complex data into more understandable, useful tools for farmers and researchers.

## 1. Introduction

The Mexican hairy pig originates from pigs that arrived in Mexico from Europe in 1492, and there are three subpopulations: Mexican Hairy, Cuino, and Mule’s Foot [1]. The largest Mexican hairy pig population is currently found in Veracruz, Puebla, Tabasco, and the Yucatan Peninsula [2]. In this region, the hairy pig is of great importance as it is part of the culture and gastronomy of the communities; hairy pig producers are creating associations specialized in the breeding of this biotype and there is a need for information that will allow them to be managed appropriately [3].

In pig production, live weight is an important indicator in determining the optimal time for slaughtering pigs, minimizing costs and labor during the fattening phase [4]. While directly measuring live weight using scales is considered one of the most accurate methods, it may not always be feasible, especially in field conditions. The lack of suitable weighing equipment in many small-scale or traditional businesses, or the economic and logistical challenges of establishing weighing systems, can limit the direct measurement of live weight. Furthermore, the need to transport or restrain animals during the weighing process can, in some cases, increase the risk of stress and injury for both animals and producers. Also, to implement the robotic technologies or visual processing methods into livestock sector, biometric studies should be conducted before [5]. Indirect methods, such as 3D imaging, offer alternatives, but more practical approaches to estimating live weight are still needed [6,7,8]. Even in the indirect methods used for animal welfare, biometric measurements should be taken by the breeders or researchers first to build up the model.

On the other hand, biometric measurements have also been used as indirect methods for predicting live weight; some of the most used morphometric measurements include chest circumference, height at withers, hip width/height, and body length. The measurements are obtained manually and used to create new estimation models for different species such as cattle [9,10,11], pigs [12,13,14,15], sheep [16,17,18], goats [19], camels [20], and yak [21]. In this study, biometric measurements were obtained manually directly from the animal using measuring instruments. Although image processing and computer vision-based methods are also widely used in the literature for obtaining biometric features, the manual measurement method was preferred in this study to offer a more applicable and practical approach in field conditions. This approach increases applicability, especially in production systems where technological infrastructure is limited.

To achieve this aim, the researchers employed various parametric statistical approaches that require strict data structure assumptions. Generally, these types of datasets cannot provide the assumptions needed [22]. To overcome these problems, several machine learning algorithms were proposed. Currently, there are a few studies that have applied machine learning techniques in animal science. Caraviello et al. [23] and Shahinfar et al. [24] analyzed production variables in dairy cattle using different machine learning techniques, including decision trees and support vector machines. Lee et al. [25] developed a deep learning-based algorithm for anomaly detection in cattle. The productivity of sows was investigated using a machine learning scheme in Lee and Choe [26]. Few studies have been conducted on estimating live weight from body measurements for different species. In this context, we aimed to predict live weight from body measurements for Yucatecan Criollo pigs using the XGBoost and LightGBM algorithms, which are the most commonly suggested algorithms in this aspect for various species.

## 2. Materials and Methods

The study area was located in the Livestock Production Units (UPP) of the cooperating producers of the project, distributed in different municipalities of the states of Campeche (San Francisco de Campeche), Yucatán (Merida), and Quintana Roo (Cancun), which make up the Yucatán Peninsula, with coordinates 19°41′34.87′ N, 89°3′1.33′ W, and a climate that varies from warm sub-humid, warm semi-warm sub-humid to dry warm semi-dry [27], according to the Centro de Investigación Científica de Yucatán, A.C. [28].

**Figure 1:**
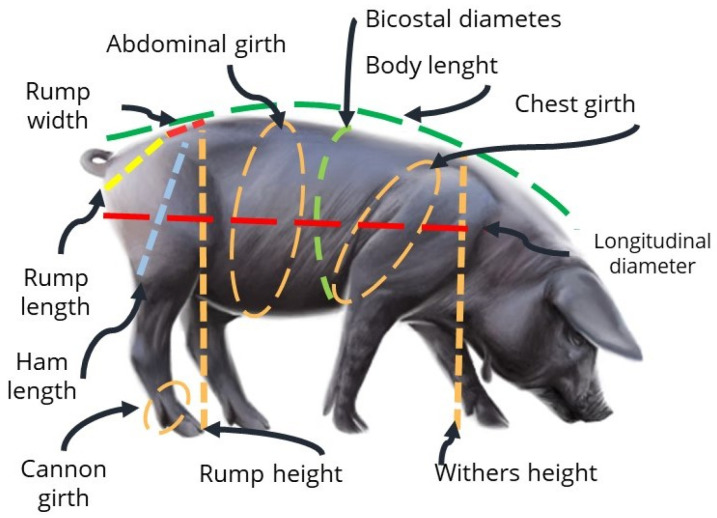
The visualization of body measurements on the pig sample.

The biometric measurements obtained were as follows (Figure 1): body weight (BW), head length (HeL), body length (BL), ham length (HaL), rump length (RL), rump height (RH), withers height (WH), chest girth (CheG), abdominal girth (AG), cannon girth (CanG), longitudinal diameter (LD), bicostal diameter (BD), and rump width (RW). All measurements were obtained manually following standard measurement procedures. Linear, girth, and diameter measurements were recorded in centimeters (cm) using a flexible measuring tape, while body weight (BW) was measured in kilograms (kg) using a digital weighing scale. During measurements, animals were kept in a natural standing position to ensure measurement consistency and reduce potential variability.

### 2.1. eXtreme Gradient Boosting Algorithm (XGBoost)

eXtreme Gradient Boosting (XGBoost), developed by Chen and Guestrin [29], is a machine learning algorithm based on gradient boosting decision trees. In this algorithm, the prediction process consists of weak learners that improve each other sequentially. XGBoost aims to increase the generalization ability of the model by adding a regularization term to the target function in order to prevent overfitting [30].

(1) $$O=\sum_{i=1}^{n}\left(L\left(y_{i},F\left(x_{i}\right)\right)\right)+\sum_{k=1}^{t}R\left(f_{k}\right)+C$$

where *O* denotes the objective function of the prediction model, *C* is a constant, and the iteration time is represented by *k*. $L\left(y_{i},F\left(x_{i}\right)\right)$ is the loss function, which measures the difference between the actual value and the estimated value for the response variable. The regularization term is represented by $R\left(f_{k}\right)$, which is shown below [30].

(2) $$R\left(f_{k}\right)=\alpha H+\frac{1}{2}\eta \sum_{j=1}^{H}w_{j}^{2}$$

where α denotes complexity parameter for the leaves, *H* denotes the number of leaves, *w_j_* denotes output of each node, and η denotes the penalty parameter [30].

The XGBoost algorithm works by creating an ensemble model consisting of a large number of decision trees trained on different subsets of the data [31]. In each decision tree, the most appropriate branching points over the features and the threshold values corresponding to these points are determined based on the depth or level of the tree. To strengthen the effect of tree structures, the model performs sequential and stepwise splits [29]. The final prediction value of the target variable is obtained by summing the prediction scores from the stable trees obtained during the training process [32].

### 2.2. A Highly Efficient Gradient Boosting Decision Tree (LightGBM)

LightGBM algorithm is a gradient boosting learning framework that uses tree-based learning methods [33] and was developed by Microsoft researchers in 2017 [34]. This algorithm has a distributed and optimized structure that provides faster training time and higher efficiency compared to other methods. LightGBM is based on methods such as gradient-based one-way sampling technique, customized feature packing, depth-limited histogram, and leaf-focused growth strategy. In addition, the LightGBM algorithm includes a regularization term in the objective function to prevent overfitting and improve the model’s generalization performance. This allows the model to minimize prediction error while simultaneously controlling model complexity [34].

(3) $$O=\sum_{i=1}^{n}L\left(y_{i},{\hat{y}}_{i}\right)+\sum_{k=1}^{t}\Omega \left(f_{k}\right)$$

where O denotes the overall objective function to be minimized during the training process, which consists of both the loss component and the regularization term. The variable n denotes the total number of observations in the dataset, while i indicates the index of each observation. The value $y_{i}$ corresponds to the actual (observed) response value for the i-th observation, and ${\hat{y}}_{i}$ represents the predicted value generated by the model. The function $L(y_{i},{\hat{y}}_{i})$ defines the loss function, which measures the discrepancy between the observed and predicted values and is commonly expressed as the squared error in regression problems. The term t refers to the total number of decision trees in the ensemble model, and $f_{k}$ denotes the k-th decision tree that contributes to the final prediction. The regularization component $\Omega (f_{k})$ is introduced to control model complexity and reduce overfitting [34].

(4) $$\Omega \left(f_{k}\right)=\gamma T+\frac{1}{2}\lambda \sum w_{j}^{2}$$

where γ is a parameter that penalizes the number of leaves in the tree, T represents the total number of leaves, λ is the L2 regularization coefficient applied to the leaf weights, and $w_{j}$ denotes the weight associated with the j-th leaf node [34].

It also aims to build a strong model by combining the ensemble of weak learners and provide high accuracy prediction performance. In particular, the histogram-based structure and maximum depth-limited leaf-focused growth approach both increase training speed and reduce memory usage [35]. In addition, the technique, which allows reaching the target leaf by reaching neighboring leaves from the main leaf during histogram extraction, increases statistical efficiency over multiple histograms by separating continuous variables into separate intervals and accelerates the convergence process [36]. In addition, LightGBM uses tree-based classifiers as a gradient boosting model [37,38,39] and iteratively constructs trees in a way that minimizes the loss function at each step. Although some methods may have limitations in terms of speed and processing power due to this structure, LightGBM can process categorical variables much more successfully than other methods, such as XGBoost [40].

In this study, which investigated the applicability of machine learning algorithms for estimating live weight of Yucatecan Criollo pigs from biometric measurements, all statistical analyses were performed using the RStudio 2026.01.1+403 [41]. The biometric measurements and sex factor were used as explanatory variables. In this context, the sex factor was evaluated as a discrete variable. Due to the imbalance in the sex distribution within the dataset (134 females and 48 males), a stratified random sampling approach was employed to divide the dataset into training and test sets, ensuring that the original sex distribution was preserved in both subsets. This ensured that the model learned to represent both sexes. In this study, separate models were not built for male and female sexes; the entire dataset was evaluated within a single model. The dataset was divided into training and testing sets with a proportion of 80:20, respectively. In addition, random seeding was used to ensure the reproducibility of the obtained results. In addition, to ensure more reliable prediction model, 10-fold cross-validation was applied to the training sets. Descriptive statistical summaries were obtained using the “psych” package [42]. The distribution of variables was assessed using the Shapiro–Wilk normality test. Since many variables did not meet the assumption of normality (*p* < 0.05), nonparametric methods were preferred for group comparisons. Differences between sexes were analyzed using the Mann–Whitney U test. False Detection Rate (FDR) correction was applied to *p*-values to control for the possibility of Type I error due to multiple comparisons. Furthermore, effect size (*r*) was calculated to assess not only statistical significance but also the magnitude of the difference. Correlation analyses were performed for the overall dataset as well as separately for sex-based subgroups (female and male). These analyses were visualized within a single framework using the GGally package in R Software [43]. To control for the increased risk of type I error due to multiple comparisons, *p*-values were adjusted using the False Discovery Rate (FDR) approach [44]. In addition to statistical significance, the magnitude of correlation coefficients was considered by evaluating effect size. For body weight estimation, the XGBoost algorithm was implemented using the “xgboost” package [45]. Additionally, the LightGBM algorithm was preferred for modeling using the “lightgbm” package [46]. The hyperparameter combinations used in each algorithm were systematically evaluated using the grid search method, and model performance for each combination was calculated using R^2^, RMSE, and MAE metrics on the training and test datasets.

## 3. Results

Table 1 presents the results of the study on estimating the live weight of Yucatecan Criollo pigs based on their biometric measurements. The table details mean, standard error, median, minimum, maximum values, and coefficients of variation (CV%) for female and male pigs. In addition, since the variables did not meet the normality assumption, comparisons between sexes were performed using the non-parametric Mann–Whitney U test; the obtained significance levels (*p*-values) and corresponding effect sizes are presented in Table 1.

According to the results presented in Table 1, differences were observed between sexes in some biometric features. According to the Mann–Whitney U test results, statistically significant differences were observed in BW, HeL, ML, CanG, and BD (*p* < 0.05). In all of these variables, the mean values of male pigs were found to be higher than those of female pigs. Effect size analysis revealed a moderate effect (*r* = 0.342) for the BW variable, while the effect size remained small in other significant variables. These results suggest that statistically significant differences may not always reflect a high level of practical or biological impact. In other biometric variables (BL, HaL, RL, RH, WH, CheG, AG, LD, and RW), no statistically significant difference was found between sexes (*p* > 0.05). In addition, according to Table 1, BW for female pigs ranged from 25 kg to 110 kg, with an average of 54.8 kg and a coefficient of 28.23%. The lowest coefficient of variation was observed in BL with 7.63%, while the highest coefficient of variation was recorded in AG with 15.29%. This shows that the biometric characteristics of the female pigs were relatively consistent. For the male pigs, BW was observed in a wide range from 34 kg to 130 kg with an average of 71.98 kg and the coefficient of variation showed a higher variability of 31.41% over 48 samples. The lowest coefficient of variation was recorded in BL with 8.84% and the highest was recorded in BD with 19.56%. The higher coefficients of variation observed in the characteristics of the male pigs suggest that the biometric characteristics show more variability in this group. In general, this table shows the biometric characteristics of Yucatecan Criollo pigs that differ according to sex and how these characteristics are distributed in the general population. It is concluded that sex has a significant effect on the biometric characteristics of pigs and that this effect should be taken into account when developing live weight estimation models. These results may allow for a more detailed examination of the sex factor in future studies and the integration of this information into pig farming practices.

**Figure 2:**
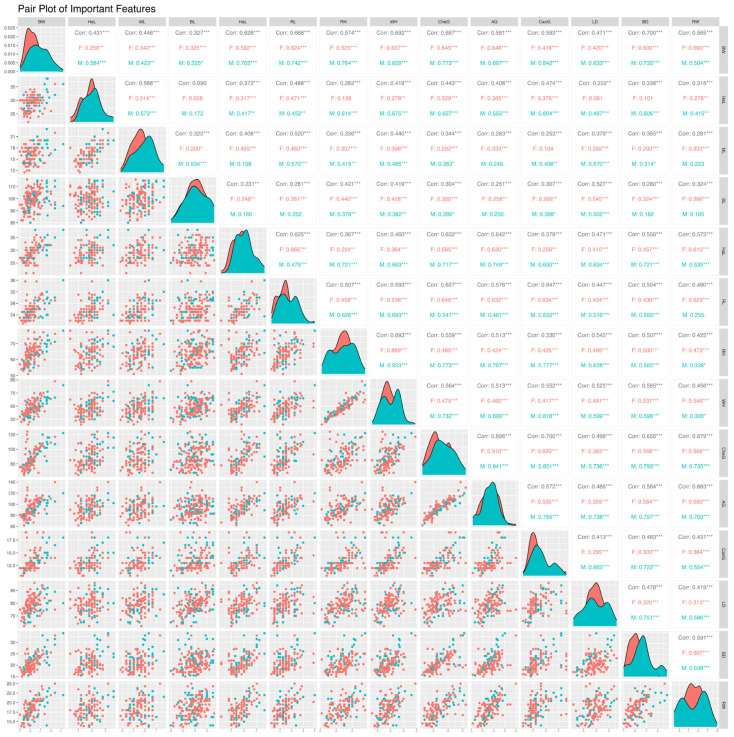
Pair-plot analysis and Pearson correlation coefficients for Yucatecan Criollo Pigs (*** = statistically significant at 0.05 level).

Figure 2 presents the relationships between biometric features of Yucatecan Criollo pigs using pair plot analysis, separated by sex. This analysis visualizes the relationships between variables and the distributions of these features for male (M) and female (F) pigs. In addition, in Figure 2, the values presented as “Corr” represent the relationships between variables calculated without regard to sex. In the color coding used in the graph, blue represents male and red represents female pigs, allowing for a clearer assessment of the direction and strength of the relationships between sexes. Relationships between features are interpreted according to the density and distribution of points in the scatter plots, with strong relationships characterized by a linear pattern and weak relationships by a more dispersed structure. The histogram and density plots on the diagonal reveal the sex-dependent distribution of each variable, providing information on the symmetry and variability of the distribution. Furthermore, to ensure statistical reliability, *p*-values were corrected using the False Discovery Rate (FDR) method, and the strength of the relationships between variables was evaluated according to effect size. The correlation structure obtained within this scope is visually presented for the entire dataset and subgroups by sex.

According to the correlation analysis results presented in Figure 2, the relationships between live weight (BW) and biometric features show significant differences both in the entire dataset and in subgroups based on sex. Almost all *p*-values obtained after FDR correction are statistically significant (mostly p_adj < 0.001), indicating that the obtained relationships are reliable despite multiple comparisons. When the entire dataset is evaluated, strong positive correlations were determined between BW and variables such as BD (r = 0.700), WH (r = 0.692), CheG (r = 0.687), and RL (r = 0.668). In contrast, the correlations of some variables such as HeL, ML, and BL with BW remained at a moderate level. In the evaluations based on sex, these relationships were found to be significantly different. For females, the correlations with BW generally range from moderate to strong, with RW (r = 0.690), CheG (r = 0.645), AG (r = 0.648), and WH (r = 0.637) variables being particularly prominent. However, it is noteworthy that the HeL variable’s correlation with BW remains weak (r = 0.256). For males, the correlation coefficients are observed to increase significantly, reaching very strong levels for many variables. In particular, CanG (r = 0.842), WH (r = 0.829), CheG (r = 0.773), RH (r = 0.764), and RL (r = 0.742) variables show very strong correlations with BW. When the entire dataset is considered, the relationships between live weight (BW) and biometric features are not only statistically significant but also strong in terms of effect size, and these relationships differ by sex. These results suggest that accounting for sex when selecting variables for live weight estimation may be important.

Table 2 presents the optimized hyperparameter value ranges for two popular machine learning models used in this study, XGBoost and LightGBM. This table shows the optimal value ranges of eta, max_depth, and min_child_weight parameters for XGBoost, and learning_rate, num_leaves, and min_data_in_leaf parameters for LightGBM, determined as a result of various experiments. In addition, the limit of the number of iterations for both models is also specified. The purpose of the table is to show the results obtained in the hyperparameter optimization process in detail and to reveal the effects of these parameters on model performance. These optimized parameters were selected to ensure that the models learn better on the data and increase the overall prediction success.

For both models, hyperparameter optimization was performed to provide the best model performance. For the XGBoost model, optimization was performed on values ranging from 0.05 to 0.3 for learning rate (eta), 2 to 10 for maximum depth, and 1 to 6 for minimum child weight. For the LightGBM model, learning rate (learning_rate) was set between 0.01 and 0.1, num_leaves between 20 and 60, and minimum data per leaf (min_data_in_leaf) between 20 and 60. These optimization processes were performed to increase the prediction success of the models, and the obtained parameter ranges play an important role in increasing the sensitivity and generalizability of the model.

Table 3 presents the optimum hyperparameter values for two popular machine learning models, XGBoost and LightGBM, and the goodness-of-fit criteria for each model. For XGBoost, the optimal hyperparameters are determined as learning rate (eta) 0.2, maximum depth (max_depth) 3, and minimum child weight (min_child_weight) 1. For LightGBM, the learning rate (learning_rate) 0.08, number of leaves (num_leaves) 20, and minimum number of data per leaf (min_data_in_leaf) 20.

**Figure 3:**
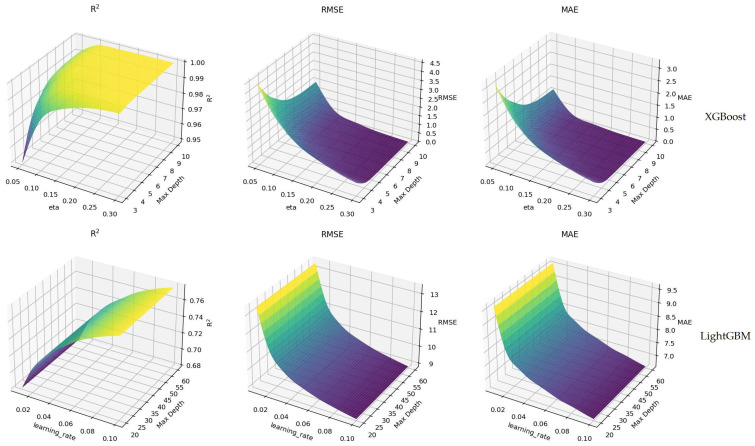
Hyperparameter optimization of XGBoost and LightGBM models (train set).

According to Table 3, the performance of both models on the training and test datasets was evaluated with R-squared (R^2^), Mean Squared Error (RMSE), and Mean Absolute Error (MAE) metrics. The R^2^ value of the XGBoost model was calculated as 0.997 in the training set and 0.905 in the test set. RMSE was determined as 1.064 in training and 5.704 in the test; MAE was determined as 0.738 in training and 3.636 in the test. The obtained performance values indicate that the model achieved a good level of fit to the training dataset and exhibited a relatively lower, yet acceptable and reliable predictive performance on the test dataset. For the LightGBM model, the R^2^ value was determined as 0.915 in the training set and 0.824 in the test set. These values show that the LightGBM model also learned the data well and can make reasonable generalizations. RMSE was measured as 5.781 in training and 7.772 in testing; MAE was measured as 4.201 in training and 5.505 in testing. Although these results show that the LightGBM model has slightly lower performance than the XGBoost model, it still reveals that it is an effective model that can be preferred depending on different data structures and features. As a result, both models achieved significant performance with their respective hyperparameter configurations, and the XGBoost model in particular attracted attention for its high R^2^ values. These results can serve as an important reference when selecting models and adjusting hyperparameters on similar datasets. Figure 3 presents visuals comparing the performances of XGBoost and LightGBM models on training sets with R^2^, RMSE, and MAE metrics.

**Figure 4:**
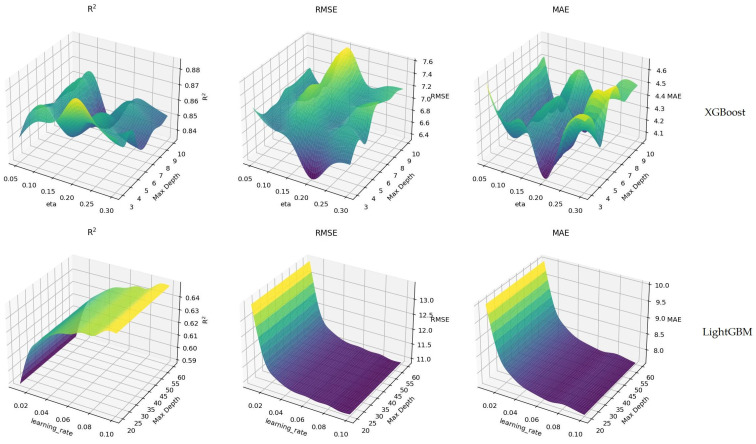
Hyperparameter optimization of XGBoost and LightGBM models (test set).

The XGBoost model generally showed improvement in R^2^ values with the increase in learning rate, reaching a maximum level of 0.99. This shows that the model explains the dataset to a high extent and provides a good fit. This situation reveals that the XGBoost model can make predictions with less error, especially at eta values and depths. On the other hand, the R^2^ values for the LightGBM model were high when the learning rate was around 0.08, but it was observed that these values became more stable as the learning rate increased. According to these results, it is shown that the LightGBM model can also provide efficient results at certain parameter settings. The performances of both models emphasize how critical the hyperparameters used and the configuration of the model are. Model selection and hyperparameter adjustments should be made carefully, considering the nature of the dataset and the specific requirements. Determining the ideal model settings can be possible by understanding the structure and characteristics of the data well and applying this information correctly. In this context, the optimization processes of both models should be considered an important step to increase the model’s overall success and make more accurate predictions. The visuals in Figure 4 show the hyperparameter optimization results of the XGBoost and LightGBM models on the test dataset. The results obtained using three different performance metrics (R^2^, RMSE, and MAE) for both models are presented comparatively.

**Figure 5:**
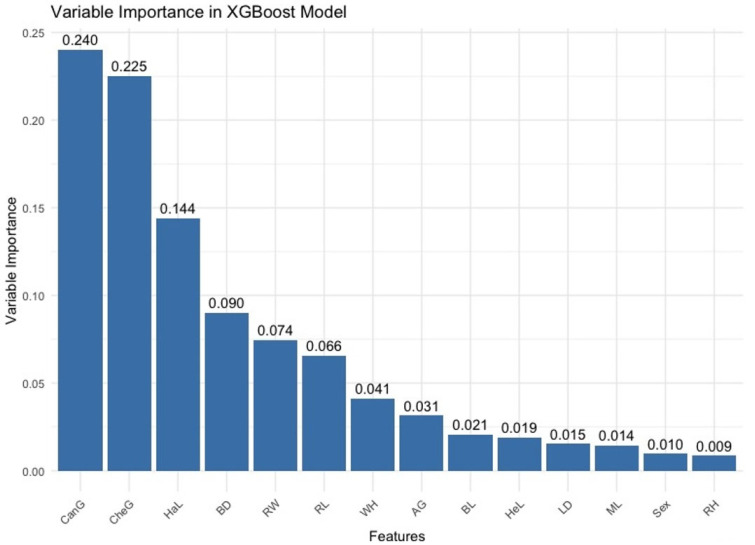
Variable importance for the XGBoost model according to sensitivity analysis.

As a general comment for the visuals in Figure 4, the performance analysis of both models on the test set clearly shows the impact of hyperparameters on model performance. The XGBoost and LightGBM models yield the best results, especially at certain combinations of learning rate and maximum depth. These figures highlight the importance of model selection and hyperparameter tuning and show the usefulness of such analyses in the model development process. In conclusion, both models can be effective in certain settings and optimization processes play a critical role in maximizing the potential of these models. Figure 5 shows the variable importance values calculated based on the sensitivity analysis results; these values belong to the XGBoost model used in body weight prediction.

**Figure 6:**
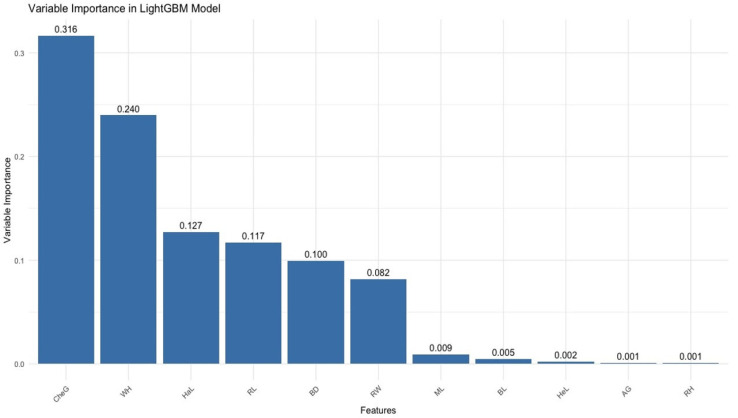
Variable importance for the LightGBM model according to sensitivity analysis.

According to Figure 5, it was statistically determined that the most important variables for body weight prediction were CanG, CheG, HaL, BD, and RW. Figure 6 shows the variable importance values calculated based on the sensitivity analysis results; these values belong to the LightGBM model used in body weight prediction. This figure identifies the features that play key roles in the model’s predictive ability and quantitatively presents the relative effects of the features on the model.

According to Figure 6, the most striking finding is that CheG has the highest importance with 0.3166, indicating that this feature has a central role in the model’s predictions. Secondly, WH stands out with a value of 0.240, indicating that it has a significant effect on the model outputs. On the other hand, HaL and RL are observed to have a significant but lower effect with 0.127 and 0.117, respectively. The other features shown in the graph contribute less to the model’s performance with much lower importance levels. This analysis allows for a better understanding of the model and, if necessary, strategic decisions regarding feature selection, while also suggesting the removal of low importance features in order to simplify the model and increase its efficiency.

**Figure 7:**
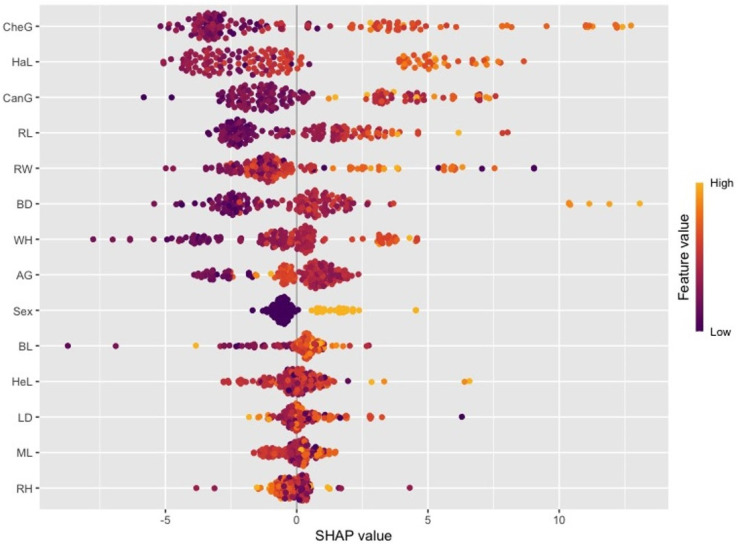
SHAP (Beeswarm) variable effects for the XGBoost model.

**Figure 8:**
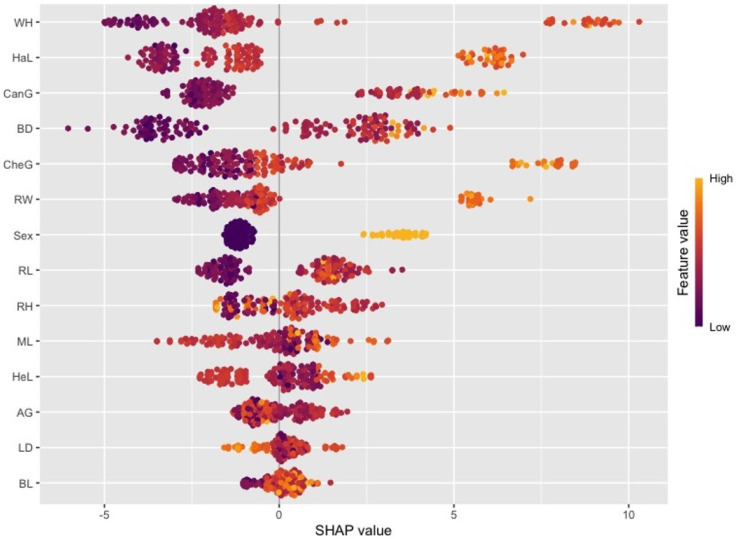
SHAP (Beeswarm) variable effects for the LightGBM model.

When Figure 7 and Figure 8 are considered, it is seen that the XGBoost and LightGBM models reveal significant differences in terms of the importance levels they attribute to the variables and the direction of the effects of these variables on the prediction. In the SHAP graph of the XGBoost model (Figure 7), it is noteworthy that the variables CheG, HaL, and CanG have wider SHAP value ranges and have a strong effect on the prediction. High values (yellow tones) in these variables are generally associated with positive SHAP values, thus contributing to an increase in live weight prediction. In contrast, variables such as BL, HeL, and RH have a narrower distribution and their contribution to the model remains relatively limited.

In the SHAP graph of the LightGBM model (Figure 8), a different distribution of variable effects is observed. In this model, the variables WH, HaL, and CanG stand out and reach higher SHAP values. Unlike the XGBoost model, the LightGBM model shows that some variables (e.g., BD, RW, and RL) have a wider range of influence in both positive and negative directions, indicating that the model captures intervariate interactions differently. Furthermore, the SHAP distributions of variables in the LightGBM model exhibit a more widespread and heterogeneous structure, which may be due to the model’s leaf-wise growth strategy and its focus on more specific data regions.

When both models are examined, it is seen that the HaL and CanG variables make significant contributions in both models, thus being among the key determinants in live weight estimation. However, it is understood that the XGBoost model yields more weight to certain variables, while the LightGBM model distributes variable effects in a more balanced and spread manner. These differences stem from the tree-building strategies (level-wise vs. leaf-wise) and learning dynamics of the two algorithms, highlighting the importance of considering data structure and intervariate relationships in model selection.

In addition, the differences in the obtained variable importance values between the XGBoost and LightGBM models examined in the current study are due to the structural differences in the data approach and learning mechanisms of these two models. XGBoost uses the gradient-boosted decision tree methodology, which tries to maximize the information gain while minimizing the error rate in each iteration. In this process, the model can provide more weight to certain features of the dataset, especially when expanding the tree branches, which causes the importance levels of some variables to increase. On the other hand, LightGBM is designed to increase efficiency and speed in large datasets as a lighter gradient-boosting model. LightGBM’s leaf-oriented growth strategy allows the model to grow more efficiently with lower losses and leads to the formation of different weights on different features. These structural and algorithmic differences have caused different feature importance levels to emerge in the examined dataset. This analysis shows that such structural differences should be taken into account in the model selection process and during the tuning of hyperparameters. In particular, understanding how models affect the learning dynamics on data and the optimization of feature selection will contribute to the development of more accurate and effective prediction models.

The findings clearly show how the structural and algorithmic differences of both models affect the importance ascribed to the features in the dataset. This analysis highlights the importance of considering the learning dynamics of the models in model selection and hyperparameter tuning, thus contributing to the development of more effective and accurate prediction models. In future studies, investigating how such structural differences perform under larger datasets and various scenarios will play a critical role in expanding the application areas of machine learning models and developing model optimization strategies. This study also provides valuable information that removing low-importance features from the model both reduces the complexity of the model and increases computational efficiency.

## 4. Discussion

The results obtained in our study show the differences in variable importance between XGBoost and LightGBM models in detail. These differences reveal how both advanced machine learning models’ hyperparameter settings and structural features affect variable importance. In addition, the findings provide valuable insights for strategic decision-making in data science and machine learning applications. The effects of these differences on model performance and the underlying reasons are discussed by providing suggestions for effective management of model selection and hyperparameter optimization processes. This analysis provides a better understanding of the models, which will contribute to more accurate and effective predictions, especially on large datasets.

The approach proposed in this study offers a practical and feasible solution for estimating live weight using biometric measurements. This method stands out as an alternative tool, especially in situations where weighing in the field is difficult, time-consuming, or poses risks to animal welfare. The developed machine learning models can provide fast and reliable predictions, support producers’ decision-making processes, and contribute to increased efficiency in herd management. Furthermore, its low cost and ease of implementation increase its potential for widespread use in various production systems. Live weight estimation studies conducted with machine learning models depend largely on the biometric characteristics of the animal species or breed to which the study is applied, as well as the success of the algorithms used. Since the genetic structure, growth pattern, body composition, and responses to environmental factors of each breed may differ, it may not be expected that the same model will show similar levels of success in different breeds or species. Therefore, the results obtained should be evaluated only in the context of the dataset and animal material used in the study. In order to use similar estimation approaches in different species or breeds, the model must be retrained, feature selection must be adapted according to the morphological and physiological structure of the relevant animal group and hyperparameters must be optimized according to the new data structure. This situation reveals that similar modeling studies should be structured specifically for each animal population and model generalizations should be made carefully.

Although advanced machine learning algorithms such as XGBoost and LightGBM have been used in the solution of various estimation and classification type problems in agriculture and animal husbandry in recent years, their areas of use are still limited, especially in specific applications such as live weight estimation. The number of studies in the literature on the application of these algorithms on animal husbandry data is quite low, and existing studies are generally limited to limited sample sizes, narrow-scope datasets, or analyses specific to certain breeds. This situation prevents the generalizability of these algorithms to be tested on different species and breeds and their wider applicability in the livestock sector. However, these algorithms offer significant advantages such as acceptable accuracy estimation capacity, resistance to over-learning, and the ability to optimize model performance through hyperparameter settings. Therefore, studies based on larger-scale and diversified datasets are needed for these methods to be more widely applied in animal breeding, especially in the estimation of economically important parameters such as live weight estimation.

In the study by Sungirai et al. [14], the relationship between linear body measurements and live weight in Landrace and Large White breed pigs was investigated and it was revealed that the model created with the linear regression method exhibited different levels of accuracy depending on the rearing conditions of the animals. While a high correlation was obtained especially in commercial farm conditions, it was reported that the accuracy level of the similar model decreased significantly under different management conditions. These findings show that live weight estimation models are sensitive not only to the variables used but also to the rearing environment and care conditions of the animals. In the present study, live weight of Yucatecan Criollo pigs was estimated with advanced machine learning algorithms such as XGBoost and LightGBM and high levels of accuracy were obtained. However, the findings of the study by Sungirai et al. [14] emphasize that such models may not be directly applicable to different breeds or different rearing systems and that model generalizability may be limited. This situation indicates that the models developed for live weight estimation are valid only within the framework of specific breeds and rearing conditions and should be adapted for different populations. Therefore, the acceptable-performance prediction models obtained in the present study should only be considered valid for pigs raised under similar environmental and management conditions.

In the study of Herrera-Camacho et al. [47], the XGBoost and LightGBM algorithms were used to body weight prediction in Holstein X Zebu crossbred heifers. According to the results of this study, it yielded the most reliable results, especially in terms of R^2^, RMSE, and MAE. In this respect, the results reported by Herrera-Camacho et al. [47] support the performance of XGBoost and LightGBM in the current study conducted for live weight estimation. When the obtained R^2^ and error measures (RMSE and MAE) are compared with the existing study, it can be said that similar results were obtained. However, it is considered that the differences observed in hyperparameter combinations may be due to variations in breeding conditions, environmental factors, and data structure among species, breeds, and farms.

In the study of Shehadeh et al. [48], modified decision tree (MDT) was determined as the model with the acceptable accuracy rate among the machine learning algorithms used for the purpose of estimating the residual value of heavy construction equipment; however, LightGBM and XGBoost algorithms also achieved successful results with high R^2^ values. While LightGBM stood out with higher accuracy than XGBoost in this study, the opposite was observed in the current study, and XGBoost showed a higher performance compared to LightGBM (R^2^ = 0.824) with R^2^ = 0.905 in the test data. This difference shows that the performance of the algorithms may vary depending on the structure of the dataset used, sample characteristics, and variable relationships. In addition, although it was stated in the study of Shehadeh et al. [48] that the MDT model yielded the best results, the lack of detailed information about the dataset limits the comparability of the results. Therefore, although similar algorithms were used in both studies, data structure and problem-specific factors may have created a difference in the model ranking. This situation reveals that the generalizability of the algorithms should be evaluated carefully.

In the study of Alabdullah et al. [49], LightGBM and XGBoost algorithms were used to predict the fast chloride permeation resistance of metakaolin, and both models were evaluated with the same performance metrics on training and test data. In that study, the LightGBM algorithm showed a more successful prediction performance compared to XGBoost. However, this situation was reversed in the current study; the XGBoost model showed a better overall performance with higher R^2^ and lower error values compared to LightGBM. These conflicting findings show that the performance of both algorithms may vary depending on many factors such as the structure of the dataset, sample characteristics, and relationships with the target variable. The fact that detailed information about the dataset was not provided in the study of Alabdullah et al. [49] limits the generalizability of comparisons between algorithms. In this context, although similar algorithms were used in both studies, it is understood that the model performance ranking may differ depending on the problem-specific data structure.

In the study conducted on rabbits by Önder et al. [50], over 95% accuracy was achieved in body weight estimation with XGBoost and LightGBM algorithms, and especially BL (body length) stood out as the most explanatory variable. In the current study, with a wider set of biometric variables belonging to Yucatecan Criollo pigs, the XGBoost model showed higher performance compared to LightGBM with R^2^ = 0.905 in the test data. Although it is seen that these algorithms are powerful tools for body weight estimation in both studies, differences in model performances may be due to the species used, variable structure and hyperparameter settings. This situation reveals that model applications should be evaluated specifically on a species and breed basis.

As the above-mentioned studies show, the use of advanced machine learning algorithms such as XGBoost and LightGBM in the field of animal husbandry, especially for live weight estimation, is extremely limited in the literature. The importance of the present study lies in the fact that it comparatively evaluates these two algorithms and makes a new contribution by successfully applying them to a unique animal material such as Yucatecan Criollo pigs. Although the acceptable-performance results obtained show that these algorithms can be used effectively in animal production data, considering the limited application examples in the literature, it is understood that such models should be evaluated separately for different breeds, species, and management systems. Therefore, the findings of the study should only be accepted as valid for animals raised under similar conditions; more empirical studies covering different environmental conditions and species-based are needed to reach wider application areas.

## 5. Conclusions

In this study, the performance of XGBoost and LightGBM models in predicting live weight in Yucatecan Criollo pigs using biometric measurements was comparatively evaluated, particularly in terms of hyperparameter optimization and variable importance levels. The results show that both models successfully captured the relationships between biometric features and live weight. However, the XGBoost model was found to exhibit superior prediction performance with higher R^2^ values and lower RMSE and MAE values. The LightGBM model, on the other hand, produced acceptable results under certain hyperparameter combinations, demonstrating that it can offer an alternative approach depending on the data structure and modeling objective. Variable importance analysis revealed that the two models assigned different levels of importance to the input variables. The XGBoost model, based on a level-wise tree growth strategy, yielded more weight to certain variables such as CanG, CheG, HaL, and BD, while the LightGBM model, in line with a leaf-wise growth strategy, highlighted variables such as CheG and WH and spread variable importance across a wider set of variables. These findings demonstrate that model selection and hyperparameter optimization should be performed considering the dataset structure and the learning mechanisms of the algorithms.

SHAP analysis, applied in addition to traditional variable significance measures, revealed the effects of variables on the model output in a more detailed and interpretable way. The SHAP results confirm that the variables HaL and CanG play a critical role in live weight estimation in both models. However, the ways in which the models captured variable effects differed. While variable effects were observed to be more pronounced and directional in the XGBoost model, variable effects exhibited a more widespread and heterogeneous distribution in the LightGBM model. This indicates that the LightGBM model can capture interactions between variables at a more local level, while the XGBoost model focuses on more dominant global patterns. From an application perspective, the approach proposed in this study offers a reliable and applicable alternative for estimating live weight based on biometric measurements. Especially in situations where weighing in the field is difficult, costly, or risky in terms of animal welfare, such models can support producers’ decision-making processes and increase operational efficiency by providing rapid and non-invasive estimates.

In conclusion, it is important to note that the findings of this study are based on a specific dataset of Yucatecan Criollo pigs. Therefore, caution should be exercised when generalizing these results to other breeds, populations, or production systems. Furthermore, the uneven sex distribution in the dataset and the model’s limitation to only biometric measurements are among the limitations that should be considered when interpreting the results. In addition, while both models exhibit acceptable performance, model success and interpretability differ depending on the data structure and the relationships between variables. Future studies are recommended to apply these approaches to larger, more diverse datasets, evaluate variable reduction strategies, and conduct research to improve the models’ generalization capabilities.

## Figures and Tables

**Table 1:** Descriptive statistics for each trait.

Variables	Sex *	Mean ± Std. Error	Median (Min–Max)	CV (%)	Sig.	Effect Size (*r*)
BW [kg]	Male	71.98 ± 3.26 ^a^	70.00 (34.00–130)	31.41	0.000	0.342
	Female	54.80 ± 1.34 ^b^	53.00 (25.00–110.00)	28.23		
HeL [cm]	Male	30.33 ± 0.45 ^a^	31.00 (26.00–38.00)	10.35	0.007	0.223
	Female	28.64 ± 0.24 ^b^	29.00 (22.00–36.00)	9.78		
ML [cm]	Male	18.03 ± 0.37 ^a^	18.50 (13.50–23.00)	14.14	0.001	0.281
	Female	16.42 ± 0.18 ^b^	16.50 (11.50–22.00)	12.79		
BL [cm]	Male	101.08 ± 1.29	101.00 (80.00–115.00)	8.84	0.477	0.071
	Female	99.66 ± 0.66	100.00 (82.00–116.00)	7.63		
HaL [cm]	Male	30.26 ± 0.47	30.00 (25.00–38.00)	10.84	0.058	0.158
	Female	28.94 ± 0.29	29.00 (21.00–36.00)	11.54		
RL [cm]	Male	26.95 ± 0.45	26.00 (22.00–36.00)	11.58	0.056	0.167
	Female	25.74 ± 0.25	26.00 (21.00–35.60)	11.11		
RH [cm]	Male	64.36 ± 0.99	65.00 (53.00–78.00)	10.63	0.714	0.043
	Female	63.95 ± 0.50	64.50 (51.00–78.00)	9.02		
WH [cm]	Male	60.38 ± 0.93	61.00 (50.00–76.00)	10.63	0.176	0.118
	Female	58.71 ± 0.48	58.00 (46.00–79.00)	9.40		
CheG [cm]	Male	93.22 ± 2.05	93.00 (68.00–122.00)	15.25	0.058	0.160
	Female	88.06 ± 1.12	85.00 (65.00–124.00)	14.66		
AG [cm]	Male	92.83 ± 1.87	96.00 (66.00–120.00)	13.98	0.828	0.025
	Female	93.83 ± 1.24	95 (62.00–140.00)	15.29		
CanG [cm]	Male	14.10 ± 0.27 ^a^	13.75 (11.00–18.00)	13.26	0.006	0.231
	Female	13.23 ± 0.15 ^b^	13 (10.50–19.00)	12.85		
LD [cm]	Male	79.23 ± 1.25	79.00 (65.00–94.00)	10.93	0.870	0.012
	Female	79.04 ± 0.54	79.00 (62.00–96.00)	7.96		
BD [cm]	Male	23.42 ± 0.66 ^a^	23.50 (15.50–34.00)	19.56	0.002	0.265
	Female	20.85 ± 0.27 ^b^	20.40 (15.00–33.00)	15.25		
RW [cm]	Male	18.83 ± 0.45	19.45 (14.00–23.80)	16.41	0.828	0.022
	Female	18.81 ± 0.20	18.60 (13.60–25.00)	12.55		

* N_Female_ = 134; N_Male_ = 48; a, b = different letters within same variables shows statistical difference between sexes (*p* < 0.05).

**Table 2:** Optimized hyperparameter value ranges for XGBoost and LightGBM models.

Optimized Hyperparameters Values for Each Model			
XGBoost		LightGBM	
eta	from 0.05 to 0.3	learning_rate	from 0.01 to 0.1
max_depth	from 2 to 10	num_leaves	from 20 to 60
min_child_weight	from 1 to 6	min_data_in_leaf	from 20 to 60

**Table 3:** Optimum hyperparameter values and goodness-of-fit criteria for both models.

Optimum Hyperparameters Values for Each Model					
XGBoost			LightGBM		
eta	0.2		learning_rate	0.08	
max_depth	3		num_leaves	20	
min_child_weight	1		min_data_in_leaf	20	
Goodness-of-fit criteria					
XGBoost	Train	Test	LightGBM	Train	Test
R^2^	0.997	0.905	R^2^	0.915	0.824
RMSE [kg]	1.064	5.704	RMSE [kg]	5.781	7.772
MAE [kg]	0.738	3.636	MAE [kg]	4.201	5.505

## Data Availability

The data are accessible from the contact author upon request.
